# Construction of a new complete growth reference for urban Chinese children

**DOI:** 10.1186/s12889-022-14702-8

**Published:** 2022-12-14

**Authors:** Wei Wu, JingNan Chen, MinJia Mo, Shuting Si, Ke Huang, RuiMin Chen, Mireguli Maimaiti, ShaoKe Chen, Chunxiu Gong, Min Zhu, ChunLin Wang, Zhe Su, Yan Liang, Hui Yao, HaiYan Wei, RongXiu Zheng, HongWei Du, Yu Yang, FeiHong Luo, Pin Li, LanWei Cui, GuanPing Dong, YunXian Yu, Junfen Fu

**Affiliations:** 1 Department of Endocrinology, The Children’s Hospital, Zhejiang University School of Medicine, National Clinical Research Center for Child Health, 3333 Binsheng Road, Hangzhou, 310052 Zhejiang China; 2 School of Public Health, Zhejiang University, 866 Yuhangtang Road, Hangzhou, 310058 Zhejiang China; 3 Department of Endocrinology, Genetics and Metabolism, Fuzhou Children’s Hospital of Fujian Medical University, Fuzhou, China; 4 Department of Endocrinology, Genetics and Metabolism, The First Affiliated Hospital of Xinjiang Medical University, Urumqi, China; 5 Department of Pediatrics, Nanning Women and Children’s Hospital, Nanning, China; 6 Department of Endocrinology, Beijing Children’s Hospital, Capital Medical University, Beijing, China; 7 Department of Endocrinology, The Children’s Hospital of Chongqing Medical University, Chongqing, China; 8 Department of Pediatrics, The First Affiliated Hospital, Zhejiang University School of Medicine, Hangzhou, China; 9 Department of Endocrinology, Shenzhen Children’s Hospital, Shenzhen, China; 10 Department of Pediatrics, Tongji Medical College of Huazhong University of Science and Technology, Wuhan, China; 11 Department of Endocrinology, Wuhan Women and Children’s Health Care Center, Wuhan, China; 12 Department of Endocrinology, Genetics and Metabolism, Zhengzhou Children’s Hospital, Zhengzhou, China; 13 Department of Pediatrics, Tianjin Medical University General Hospital, Tianjin, China; 14 Department of Pediatrics, The First Bethune Hospital of Jilin University, Jilin, China; 15 Department of Endocrinology, Jiangxi Provincial Children’s Hospital, Nanchang, China; 16 Department of Endocrinology, Genetics and Metabolism, Children’s Hospital of Fudan University, Shanghai, China; 17 Department of Endocrinology, Children’s Hospital of Shanghai, Shanghai, China; 18 Department of Pediatric, The First Affiliated Hospital of Harbin Medical University, Harbin, China

**Keywords:** Growth reference, Urban Chinese children, Height-for-age, Weight-for-age

## Abstract

**Background:**

Growth chart is a valuable clinical tool to monitor the growth and nutritional status of children. A growth chart widely used in China is based on the merged data sets of national surveys in 2005. We aimed to establish an up-to-date, complete growth curve for urban Chinese children and adolescents with a full range of ages.

**Methods:**

Using data collected in a large-scale, cross-sectional study (Prevalence and Risk factors for Obesity and Diabetes in Youth (PRODY), 2017–2019), we analyzed 201,098 urban children aged 3 to 18 years from 11 provinces, autonomous regions, and municipalities that are geographically representative of China. All participants underwent physical examinations. Sex-specific percentiles of height-for-age and weight-for-age were constructed by Generalized Additive Models for Location Scale and Shape (GAMLSS) model. We also compared the median values of height-for-age or weight-for-age between our growth chart and the established growth reference using Welch-Satterthwaite T-Test.

**Results:**

Consistent with the established growth reference, we observed that the P_50_ percentile of height-for-age reached plateaus at the age of 15 years (172 cm) and 14 years (160 cm) for boys and girls, respectively. In addition, boys aged 10 ~ 14 years and girls aged 10 ~ 12 years exhibited the most dramatic weight difference compared to those of other age groups (19.5 kg and 10.3 kg, respectively). However, our growth chart had higher median values of weight-for-age and height-for-age than the established growth reference with mean increases in weight-for-age of 1.36 kg and 1.17 kg for boys and girls, respectively, and in height-for-age of 2.9 cm and 2.6 cm for boys and girls, respectively.

**Conclusions:**

Our updated growth chart can serve as a reliable reference to assess the growth and nutritional status in urban Chinese children throughout the entire childhood.

**Supplementary Information:**

The online version contains supplementary material available at 10.1186/s12889-022-14702-8.

## Introduction

Growth and nutritional status in children and adolescents are widely considered to be important indicators of health during adulthood because of their links with a range of adverse health consequences[1]. Both under- and over-nutritional status during childhood are associated with a range of non-communicable diseases throughout life[2]. Stunting is a known risk factor to impaired child development, high fatality rate and many other serious consequences[3]. Similarly, increase in body mass index (BMI) is a major contributor to poor physical fitness and to cardiovascular risk in adolescents[2, 4].

Regular growth monitoring of children and adolescents is an important way to assess children's nutritional status and to detect diseases at early stages. Growth charts are widely used as a clinical tool to monitor growth in individual children and as a public health indicator to assess the nutritional status of a population[5–8]. Two well-known growth charts were released by the U.S. CDC [9] in May 2000 and by the WHO[10] in April 2006. However, problems may occur when these growth charts are applied to populations whose ethnicities differ from those of the reference population (e.g., Chinese children)[11].

A growth chart widely used in China is based on the merged data sets of two authoritative series of national surveys in 2005, the National Survey on the Physical Growth and Development of Children in the Nine Cities of China (NSPGDC) and the Chinese National Survey on Student’s Constitution and Health (CNSSCH)[11]. NSPGDC has conducted 5 survey waves (every 10 years since 1975) in 9 cities in mainland China. Theses waves have provided the first nationally representative sample of infants and pre-school children under 7 years in China. CNSSCH has been carried out every 5 years during 1985 ~ 2010 and in 2014 to produce a national representative sample of school-age children and adolescents (aged 7 ~ 22 years) from both urban and rural areas in 31 provinces, autonomous regions and municipalities of China. However, both surveys covered incomplete age ranges, and they differed in sampling procedures and methods of data collection. Thus, the merged data sets of these two surveys were inherently broken and artificially reconnected at the age of 6 ~ 7 years, although the authors claimed a smooth transition in the growth curve[11]. Therefore, it is imperative to establish a complete growth curve for Chinese children and adolescents with a full range of ages.

Using data collected in a large-scale, cross-sectional nationwide study (Prevalence and Risk factors for Obesity and Diabetes in Youth (PRODY)), we aimed to develop up-to-date, sex-specific percentiles of height-for-age and weight-for-age in urban Chinese children and adolescents with a full range of ages (3 ~ 18 years).

## Methods

### Study design

The details of our cross-sectional survey (Prevalence and Risk factors for Obesity and Diabetes in Youth (PRODY) from January 1, 2017 to December 31, 2019) were described previously[12]. Briefly, using a multistage, stratified, cluster-sampling design (Figure S1), a sample of urban Chinese children and adolescents was collected from non-rural areas of 11 provinces, autonomous regions, and municipalities, which had different levels of urbanization and economic development[13] (Table S1) and were representative of western, southern, eastern, northern, and central China. The study protocol was approved by the ethics review committee of The Children’s Hospital of the Zhejiang University (Approval Number: 2016-JRB-018) and the cooperating institutions. Written informed consent from participants and their guardians was obtained (children under 5 years old were allowed to draw symbols instead of a signature). All methods in our study were carried out in accordance with relevant guidelines and regulations.

### Data collection

IN the PRODY study, demographic information (e.g., name, sex, school grade, and date of birth) and medical history of participants were collected through interviews. Age was calculated as the difference between the date of birth and date of data collection. The anthropometric measurements of participants (weight and height) were taken by physicians, as described previously[12]. Height and weight outliers were defined as measurements that fell outside the median ± fourfold standard deviations (Table S2)[14].

### Statistical methods

Sex-specific percentiles of height-for-age and weight-for-age were constructed by Generalized Additive Models for Location Scale and Shape (GAMLSS) model[15], which usually contains the following parameters: position (such as the mean and median, etc.), scale (such as standard deviation, mean square deviation, coefficient of variation, etc.), the shape of the distribution, (such as skewness and kurtosis). We computed the percentiles (P_3_, P_10_, P_25_, P_50_, P_75_, P_90_, P_97_) of weight-for-age and height-for-age based on the Box-Cox Cole and Green distribution. We compared the median values of height-for-age or weight-for-age between the established growth reference for Chinese children and the growth chart based on our PRODY study using Welch-Satterthwaite T-Test. P < 0.05 was considered to be statistically significant. All of our statistical analyses were performed using R software (Version 3.5.1; R Foundation for Statistical Computing, Vienna, Austria) with the GAMLSS package.

## Results

A total sample of 201,098 healthy children and adolescents (105,875 boys [52.6%] and 95,223 girls [47.4%]; mean [SD] age of 9.8 [3.8] years) from eastern, southern, northern, central and western China was included in our analysis[12]. The recruitment flow chart was shown in Figure S2.

### Complete growth reference for urban Chinese children based on PRODY study

**Fig. 1:**
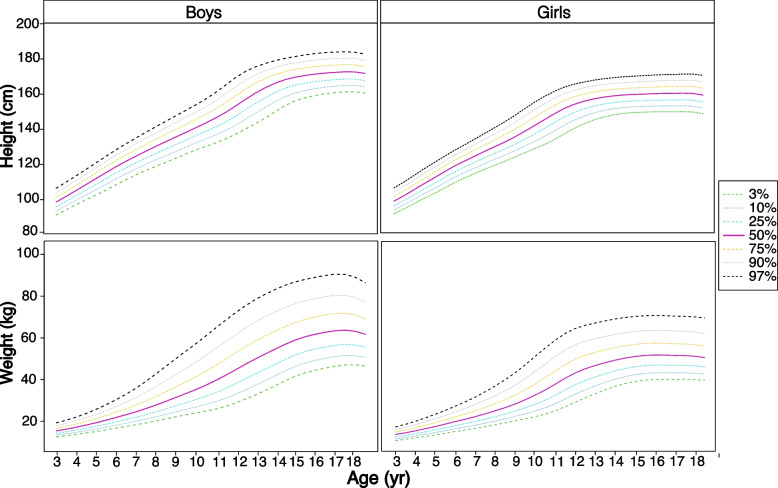
Percentiles of height-for-age and weight-for-age in the PRODY study (2017–2019). Notes: PRODY, Prevalence and Risk factors for Obesity and Diabetes in Youth

The percentiles of height-for-age and weight-for-age in the PRODY study were illustrated in Fig. 1. The P_50_ percentile of height-for-age for boys increased linearly from 3 to 14 years age (from 98.6 cm to 166.3 cm, respectively) and reached the plateau at round 172 cm by the age of 15 years. Similarly, the P_50_ percentile of height-for-age for girls also increased linearly between 3 and 13 years of age (from 97.4 cm to 157 cm, respectively) and plateaued at about 160 cm at the age of 14 years. The P_50_ percentile of weight-for-age for boys between the ages of 3 and 17 years increased from 15.4 kg to 63.4 kg. Boys in the age group of 10 ~ 14 years exhibited the most dramatic weight difference (19.5 kg) compared to those of other age groups. In contrast, the P_50_ percentile of weight-for-age for girls increased significantly between the ages of 3 to 14 years from 14.9 kg to 49.6 kg, and girls in the age group of 10 ~ 12 years exhibited the most dramatic weight difference (10.3 kg) compared to those of other age groups.

### Comparison of the growth reference based on PRODY study and the established growth reference

We further compared the growth chart based on our PRODY study with the established growth reference based on the merged data sets of the NSPGDC (2005) and the CNSSCH (2005)[11] (Table 1). We found that our growth chart had higher median values of weight-for-age and height-for-age than the established growth reference with mean increases in weight-for-age of 1.36 kg and 1.17 kg for boys and girls, respectively, and in height-for-age of 2.9 cm and 2.6 cm for boys and girls, respectively. Specifically, the median values of weight-for-age for boys (3 ~ 18 years) and girls aged 3 ~ 17 years in our growth chart were significantly higher than the established growth reference. The differences in median values of weight-for-age between these two growth references were notably large among boys aged 10 ~ 18 years (1.48 ~ 2.74 kg) and girls aged 10 ~ 16 years (1.28 ~ 2.88 kg). In addition, our growth chart had significantly higher median values of height-for-age than the established growth reference among boys aged 3 ~ 15 years and girls aged 3 ~ 14 years (increases of 1.09 ~ 5.00 cm and 1.13 ~ 4.78 cm for boys and girls, respectively), whereas the median values of height-for-age for older adolescents in these two growth references were very close.

**Table 1:** Comparison of the median values of height and weight in Chinese growth references

Age (years)	Boy			Girl		
	Established growth references for Chinese children	Growth references based on PRODY	P value	Established growth references for Chinese children	Growth references based on PRODY	P value
	Weight (kg)					
3	14.65	15.40	< 0.05	14.13	14.86	< 0.05
4	16.64	17.17	< 0.05	16.17	16.57	< 0.05
5	18.98	19.31	< 0.05	18.26	18.61	< 0.05
6	21.26	21.85	< 0.05	20.37	20.92	< 0.05
7	24.06	24.57	< 0.05	22.64	23.21	< 0.05
8	27.33	27.80	< 0.05	25.25	25.95	< 0.05
9	30.46	31.44	< 0.05	28.19	29.16	< 0.05
10	33.74	35.22	< 0.05	31.76	33.27	< 0.05
11	37.69	39.59	< 0.05	36.10	38.37	< 0.05
12	42.49	44.76	< 0.05	40.77	43.65	< 0.05
13	48.08	49.97	< 0.05	44.79	47.10	< 0.05
14	53.37	54.71	< 0.05	47.83	49.64	< 0.05
15	57.08	59.00	< 0.05	49.82	51.37	< 0.05
16	59.35	61.74	< 0.05	50.81	52.09	< 0.05
17	60.68	63.42	< 0.05	51.20	51.89	< 0.05
18	61.40	63.12	< 0.05	51.41	51.51	N.S
	Height (cm)					
3	97.5	98.6	< 0.05	96.3	97.4	< 0.05
4	100.6	105.1	< 0.05	99.4	104.0	< 0.05
5	107.7	111.9	< 0.05	106.7	110.9	< 0.05
6	114.7	118.7	< 0.05	113.5	117.7	< 0.05
7	120.7	124.6	< 0.05	119.4	123.4	< 0.05
8	127.1	130.3	< 0.05	125.6	128.9	< 0.05
9	132.7	135.6	< 0.05	131.3	134.5	< 0.05
10	137.9	140.9	< 0.05	137.0	141.2	< 0.05
11	142.6	146.4	< 0.05	143.3	148.1	< 0.05
12	148.4	153.2	< 0.05	149.7	153.7	< 0.05
13	155.6	160.6	< 0.05	154.6	157.0	< 0.05
14	163.0	166.3	< 0.05	157.6	158.8	< 0.05
15	168.2	169.7	< 0.05	159.4	159.7	N.S
16	171.0	171.4	N.S	160.1	160.1	N.S
17	172.1	172.4	N.S	160.2	160.3	N.S
18	172.5	172.6	N.S	160.5	160.2	N.S

Data source of the established growth reference for Chinese children: Zong XN et al. PloS one 2013; 8(3): e59569. PRODY, Prevalence and Risk factors for Obesity and Diabetes in Youth. N.S represented the non-significant difference between the established growth reference and the growth chart based on PRODY

## Discussion

The present study provided an up-to-date growth curve for urban Chinese children and adolescents aged 3 ~ 18 years, which can be used to estimate growth, adiposity and metabolic risk. Country-specific growth references are reported to have been more likely to describe children’s growth more veritably and faithfully than the WHO standards[16]. Therefore, it is essential to establish country-specific growth charts to improve the monitoring of growth of children. The well-established growth reference for Chinese children, which was based on data from two ongoing national representative surveys (the NSPGDC and the CNSSCH in 2005), possess a potential defect (discontinuity) in their growth curves at the age of 6 ~ 7 years. In contrast, our PRODY study collected a large-scale nationwide sample of urban children aged 3 ~ 18 years, which inherently resulted in a smooth transition throughout the full range of ages.

Consistent with the previously established growth reference[11], the P_50_ percentile of height-for-age in our growth chart based on the PRODY study, reached plateaus at the age of 15 years and 14 years for boys and girls, respectively. In addition, we found that boys aged 10 ~ 14 years and girls aged 10 ~ 12 years exhibited the most dramatic weight difference compared to those of other age groups, which might be associated with onset of puberty[17].

The differences between the growth chart based on our PRODY study and the well-established growth reference for Chinese children might be partly due to the non-representativeness of the urbanization levels. In addition, we also partly attributed the differences to secular sources of variation. These differences were supported by a previous study that analyzed successive CNSSCH data and reported that during 2005 ~ 2014 the mean increase in weight and height of urban Chinese children to be 2.6 kg and 1.6 cm, respectively[13]. Interestingly, we found that teenagers (especially boys aged 10 ~ 18 years and girls aged 10 ~ 16 years) had notably large differences in median values of weight-for-age between our growth chart and the established growth reference. Consistently, a study that analyzed successive data of children aged 6 ~ 18 years in Hong Kong revealed similar age-dependent changes in the mean weight-for-age from 1963, 1993, to 2005/6[18]. With the development of urbanization in China, children aged 6 ~ 17 years have suffered from higher prevalences of overweight and obesity than those under 6 years old since 1992[19], which supports the increase in the mean weight-for-age for older children. However, the median values of height-for-age in our growth chart only significantly differed from the established growth reference among boys aged 3 ~ 15 years and girls aged 3 ~ 14 years but not among older adolescents. In consistent with our observations, a previous study showed a similar change in height-for-age for children living in Beijing, China between 1985 and 2010[20]. We speculated that the secular change in height-for-age was different among children in different age groups might because some factors attributing to the secular change in child’s height were associated with age. For example, protein intake, which has increased with the economic development in recent years, was positively associated with height-for-age Z score only in 5 ~ 12 year group of children[21].

The strengths of our study included large sample size and broad geographical coverage, which allowed us to assess the growth of urban Chinese children over the full range of ages. In addition, the PRODY survey included weight and height measured by physicians, resulting in more accurate estimates than self-reported or parental-reported data. In contrast to the CNSSCH study implemented by different groups with different equipment, our survey was conducted by our team members using the same equipment. Given the height differences between the morning and evening, we took all the measurements in the morning. These contributed to the consistency in the measurements of weight and height. Our study had several limitations. First, given the inherent limitations of cross-sectional survey data, we could not describe the growth curve estimated in our study as the average of longitudinal changes within an individual. Second, our sampling was restricted to suburban and urban areas (91% of schools were surveyed from urban areas) because it was difficult to access the population in rural areas. Therefore, more efforts should be devoted to sampling the rural population in future studies.

In conclusion, the study provided an updated growth chart for urban Chinese children and adolescents aged 3 ~ 18 years, which can serve as a reliable reference to assess the growth and nutritional status in Chinese children throughout the entire childhood.

## Supplementary Information

**Additional file 1.**

## Data Availability

De-identified cross-sectional data of the PRODY study (2017–2019) used in the analysis can be made available after corresponding author's review of request for data.
