# Supplementary material for: Construction of a new complete growth reference for urban Chinese children
Source: BMC Public Health. 2022 Dec 14;22:2345. doi: 10.1186/s12889-022-14702-8 (PMC9749202; doi:10.1186/s12889-022-14702-8)
Supplement: Supplementary file 1 — Additional file 1. [file 12889_2022_14702_MOESM1_ESM.docx]

**Figure S1. Sampling procedure**

**Figure S2. Recruitment flow chart, Prevalence and Risk factors for Obesity and Diabetes in Youth (PRODY) study, 2017-2019**

**Table S1. Levels of urbanization and economic development of provinces, autonomous regions, and municipalities in China (2016)**

**Table S2. Median values and standard deviations of height and weight for Chinese children**

**Figure S1. Sampling procedure**

#### Note: We did not indicate the numbers of kindergartens, primary schools, middle schools and high schools surveyed in this figure because it was difficult to identify the school type for some schools spanning several school types.

####

**Figure S2. Recruitment flow chart, Prevalence and Risk factors for Obesity and Diabetes in Youth (PRODY) study, 2017-2019**

**Table S1. Levels of urbanization and economic development of provinces, autonomous regions, and municipalities in China** **(2016)**

|  | **Provinces** | **Regional gross domestic product (100 million Yuan)** | | **Urbanization ratio (%)** |
| --- | --- | --- | --- | --- |
| North | Beijing | 23014.59 | 86.5 | |
|  | Tianjin | 16538.19 | 82.64 | |
|  | Hebei | 29806.11 | 51.33 | |
|  | Shanxi | 12766.49 | 55.03 | |
|  | Inner Mongolia Autonomous Region | 17831.51 | 60.30 | |
|  | Liaoning | 28669.02 | 67.35 | |
|  | Jinlin | 14063.13 | 55.31 | |
|  | Heilongjiang | 15083.67 | 58.80 | |
| East | Shanghai | 25123.45 | 87.60 | |
|  | Jiangsu | 70116.38 | 66.52 | |
|  | Zhejiang | 42886.49 | 65.80 | |
|  | Anhui | 22005.63 | 50.50 | |
|  | Fujian | 25979.82 | 62.60 | |
|  | Jiangxi | 16723.78 | 51.62 | |
|  | Shandong | 63002.23 | 57.01 | |
| Central | Henan | 37002.16 | 46.85 | |
|  | Hubei | 29550.19 | 56.85 | |
|  | Hunan | 28902.21 | 50.89 | |
| South | Guangdong | 72812.55 | 68.71 | |
|  | Guangxi Zhuang Autonomous Region | 16803.12 | 47.06 | |
|  | Hainan | 3702.76 | 55.12 | |
| West | Chongqing | 15717.27 | 60.94 | |
|  | Sichuan | 30053.10 | 47.69 | |
|  | Guizhou | 10502.56 | 42.01 | |
|  | Yunnan | 13619.17 | 43.33 | |
|  | Xizang | 1026.39 | 27.74 | |
|  | Shananxi | 18021.86 | 53.92 | |
|  | Gansu | 6790.32 | 43.19 | |
|  | Qinghai | 2417.05 | 50.30 | |
|  | Ningxia | 2911.77 | 55.23 | |
|  | Xinjiang Autonomous Region | 9324.8 | 47.23 | |

Notes: Urbanization ratio is expressed as the proportion of urban and rural populations based on the recorded household registration system in the government’s Statistical Yearbook (2016). The underlined regions are surveyed in PRODY study.

**Table S2. Median values and standard deviations of height and weight for Chinese children**

| Age | Sex | Median height/cm | Height SD/cm | Median weight/Kg | Weight SD/Kg |
| --- | --- | --- | --- | --- | --- |
| 3.0 | Female | 95.6 | 3.8 | 14.1 | 1.7 |
| 3.5 | Female | 99.4 | 3.9 | 15.2 | 1.9 |
| 4.0 | Female | 103.1 | 3.9 | 16.2 | 2.0 |
| 4.5 | Female | 106.7 | 4.2 | 17.2 | 2.2 |
| 5.0 | Female | 110.2 | 4.3 | 18.3 | 2.4 |
| 5.5 | Female | 113.5 | 4.5 | 19.3 | 2.7 |
| 6.0 | Female | 116.6 | 4.6 | 20.4 | 2.9 |
| 6.5 | Female | 119.4 | 4.9 | 21.4 | 3.2 |
| 7.0 | Female | 122.5 | 5.1 | 22.6 | 3.5 |
| 7.5 | Female | 125.6 | 5.2 | 23.9 | 3.9 |
| 8.0 | Female | 128.5 | 5.4 | 25.3 | 4.3 |
| 8.5 | Female | 131.3 | 5.6 | 26.7 | 4.8 |
| 9.0 | Female | 134.1 | 5.8 | 28.2 | 5.3 |
| 9.5 | Female | 137.0 | 6.1 | 29.9 | 6.0 |
| 10.0 | Female | 140.1 | 6.3 | 31.8 | 6.7 |
| 10.5 | Female | 143.3 | 6.5 | 33.8 | 7.4 |
| 11.0 | Female | 146.6 | 6.7 | 36.1 | 8.0 |
| 11.5 | Female | 149.7 | 6.6 | 38.4 | 8.5 |
| 12.0 | Female | 152.4 | 6.4 | 40.8 | 8.8 |
| 12.5 | Female | 154.6 | 6.2 | 42.9 | 8.9 |
| 13.0 | Female | 156.3 | 6.0 | 44.8 | 8.8 |
| 13.5 | Female | 157.6 | 5.8 | 46.4 | 8.6 |
| 14.0 | Female | 158.6 | 5.7 | 47.8 | 8.3 |
| 14.5 | Female | 159.4 | 5.5 | 49.0 | 8.1 |
| 15.0 | Female | 159.8 | 5.5 | 49.8 | 7.9 |
| 15.5 | Female | 160.1 | 5.5 | 50.5 | 7.7 |
| 16.0 | Female | 160.1 | 5.4 | 50.8 | 7.6 |
| 16.5 | Female | 160.2 | 5.4 | 51.1 | 7.6 |
| 17.0 | Female | 160.3 | 5.4 | 51.2 | 7.5 |
| 17.5 | Female | 160.3 | 5.4 | 51.2 | 7.5 |
| 18.0 | Female | 160.6 | 5.3 | 51.4 | 7.5 |
| 18.5 | Female | 160.6 | 5.3 | 51.4 | 7.5 |
| 3.0 | Male | 96.8 | 3.9 | 14.7 | 1.7 |
| 3.5 | Male | 100.6 | 3.9 | 15.6 | 1.9 |
| 4.0 | Male | 104.1 | 4.1 | 16.6 | 2.0 |
| 4.5 | Male | 107.7 | 4.2 | 17.8 | 2.2 |
| 5.0 | Male | 111.3 | 4.4 | 19.0 | 2.5 |
| 5.5 | Male | 114.7 | 4.5 | 20.2 | 2.8 |
| 6.0 | Male | 117.7 | 4.7 | 21.3 | 3.1 |
| 6.5 | Male | 120.7 | 4.9 | 22.5 | 3.4 |
| 7.0 | Male | 124.0 | 5.1 | 24.1 | 4.0 |
| 7.5 | Male | 127.1 | 5.3 | 25.7 | 4.6 |
| 8.0 | Male | 130.0 | 5.5 | 27.3 | 5.2 |
| 8.5 | Male | 132.7 | 5.7 | 28.9 | 5.9 |
| 9.0 | Male | 135.4 | 5.8 | 30.5 | 6.5 |
| 9.5 | Male | 137.9 | 6.1 | 32.1 | 7.0 |
| 10.0 | Male | 140.2 | 6.2 | 33.7 | 7.6 |
| 10.5 | Male | 142.6 | 6.5 | 35.6 | 8.1 |
| 11.0 | Male | 145.3 | 6.8 | 37.7 | 8.6 |
| 11.5 | Male | 148.4 | 7.0 | 40.0 | 9.2 |
| 12.0 | Male | 151.9 | 7.5 | 42.5 | 9.8 |
| 12.5 | Male | 155.6 | 7.7 | 45.1 | 10.4 |
| 13.0 | Male | 159.5 | 7.8 | 48.1 | 11.0 |
| 13.5 | Male | 163.0 | 7.5 | 50.9 | 11.3 |
| 14.0 | Male | 165.9 | 7.2 | 53.4 | 11.5 |
| 14.5 | Male | 168.2 | 6.8 | 55.4 | 11.4 |
| 15.0 | Male | 169.8 | 6.5 | 57.1 | 11.3 |
| 15.5 | Male | 171.0 | 6.3 | 58.4 | 11.1 |
| 16.0 | Male | 171.6 | 6.2 | 59.4 | 10.9 |
| 16.5 | Male | 172.1 | 6.1 | 60.1 | 10.7 |
| 17.0 | Male | 172.3 | 6.1 | 60.7 | 10.5 |
| 18.0 | Male | 172.7 | 6.0 | 61.4 | 10.3 |

Notes: SD, standard deviation; Data source: Department of Maternal and Child Health and Community Health, Ministry of Health, People's Republic of China; Coordinating Study Group of Nine Cities on the Physical Growth and Development of Children; Capital institute of pediatrics. Growth standards and growth charts for Chinese children. Second Military Medical University Press; 2009.
